# All-cause mortality in patients with long-term opioid therapy compared with non-opioid analgesics for chronic non-cancer pain: a database study

**DOI:** 10.1186/s12916-020-01644-4

**Published:** 2020-07-15

**Authors:** Winfried Häuser, Tino Schubert, Tobias Vogelmann, Christoph Maier, Mary-Ann Fitzcharles, Thomas Tölle

**Affiliations:** 1 Internal Medicine 1, Innere Medizin 1, Klinikum Saarbrücken GmbH, Winterberg 1, 66119 Saarbrücken, Germany; 2 Department Psychosomatic Medicine and Psychotherapy, Technische Universität München, 81675 Munich, Germany; 3 LinkCare GmbH, 70469 Stuttgart, Germany; 4 University Hospital of Pediatrics and Adolescent Medicine, Ruhr-University Bochum, 44801 Bochum, Germany; 5 Alan Edwards Pain Management Unit, Division of Rheumatology, McGill University Health Centre, Montreal, Quebec H3G 1A4 Canada; 6 Department Neurology, Technische Universität München, 81675 Munich, Germany

**Keywords:** Long-term opioid therapy, Non-opioid analgesics, All-cause mortality, Case-control study, General population, Healthcare claims database

## Abstract

**Background:**

Hitherto only studies with selected populations have found an increased all-cause mortality of some selected opioids compared to selected non-opioids for chronic non-cancer pain (CNCP). We have examined the all-cause mortality for CNCP associated with all established opioids compared to non-opioid analgesic therapy (anticonvulsants, antidepressants, dipyrone, non-steroidal agents).

**Methods:**

The study used the InGef (Institute for Applied Health Research Berlin) database which is an anonymized healthcare claims database including 4,711,668 insured persons who were covered by 61 German statutory health insurances between 2013 and 2017.The health insurance companies are the owners of the database. All-cause mortality was determined from death certificates. Adjusted hazard ratios (HRs) including age, gender, comorbidity index, and propensity score as covariates and risk differences (RD) in incidence of death between patients with long-term opioid therapy (LTOT) and control-drug therapy were calculated.

**Results:**

The mean age of participants was 66 years; 55% were women. There were 554 deaths during 10,435 person-years for the LTOT patients, whereas there were 340 deaths during 11,342 person-years in the control group. The HR for all-cause mortality was 1.59 (95% CI, 1.38–1.82) with a risk difference of 148 excess deaths (95% CI 99–198) per 10,000 person-years. The elevated risk of death for LTOT was confined to the out-of-hospital deaths: LTOT patients had 288 out-of-hospital deaths during 10,435 person-years (276 per 10,000 person-years) whereas there were 110 deaths during 11,342 person-years (97 per 10,000 person-years) in the control group. HR was 2.29 (95% CI 1.86, 2.83). Although our propensity score matching model indicated a good classification, residual confounding cannot be fully excluded. The opioid group had a higher prevalence of heart failure and a higher use of anti-thrombotic and antiplatelet agents and of psycholeptics.

**Conclusions:**

LTOT for CNCP compared to non-opioid analgesics was associated with an increased risk for all-cause mortality. When considering treatment options for patients with CNCP, the relevant risk of increased all-cause mortality with opioids should be discussed.

**Trial registration:**

ClinicalTrials.gov, NCT03778450, Registered on 7 December 2018

## Background

Although European countries are distanced from the opioid crisis in North America [1–4], there are increasing concerns about the safety of long-term opioid therapy (LTOT) for patients with chronic non-cancer pain (CNCP) [5]. All-cause mortality has been reported to be increased in recent studies of patients on opioid therapy compared to propensity score-matched non-opioid-treated patients in selected populations: An US study compared some long-acting opioids (morphine, oxycodone, transdermal fentanyl, methadone) for CNCP with anticonvulsants and antidepressants in Medicaid enrollees < 75 years with CNCP [6]. Zeng et al. compared tramadol with non-steroidal anti-inflammatory drugs (NSAIDs) in patients with osteoarthritis aged over 50 years in a British general practitioner database [7]. Burr et al. compared weak and strong opioids versus no opioids in patients with inflammatory bowel diseases using a database of both primary and secondary care in England [8]. To date, no study has examined the mortality rate for patients with CNCP conditions treated with opioids versus all established non-opioid analgesics in a sample that is representative of the general population.

Guidelines for opioid use for CNCP recommend a shared decision-making process to provide information that allows for an informed treatment choice that balances the potential benefits and harms of individual treatments [9, 10]. Similar to opioids, alternative drug treatments for CNCP such as NSAIDs can be associated with potential serious adverse effects, e.g., cardiovascular and gastrointestinal events [11, 12]. Randomized controlled trials specifically comparing the safety of opioids to other analgesics are few in number, emphasizing the need for cohort studies based on patient registries to provide long-term safety data for drug therapy [13, 14]. This retrospective database study has compared the risk of all-cause mortality among patients initiating LTOT for CNCP with that for matched patients initiating therapy with other analgesics (anticonvulsants, antidepressants, dipyrone, NSAIDs) in a sample representative of the general population in a country without an opioid crisis [15].

## Methods

The study is reported according to the STROBE guideline [16] (Additional file 1, Table 1: Strobe Checklist).

### Data source

This study used the InGef (Institute for Applied Health Research Berlin) database which is an anonymized healthcare claims database with longitudinal data over a look-back period of up to 6 years. The database included 4,711,668 insured persons who were covered by 61 German statutory health insurances between 2013 and 2017. Claims data are transferred directly from the healthcare providers to a specialized data center owned by the health insurance companies, which provides data warehouse and information technology services. These claims data are regularly audited by the insurance companies for reimbursement purposes and are prepared in accordance with German Social Law (paragraphs 287 SGB V and 75 SGB X). In the data center, data are anonymized before entering the InGef database. Data are anonymized with respect to individual insurant, healthcare providers (e.g., physicians, practices, hospitals, and pharmacies), and the respective health insurance. Data were adjusted to Germany’s age and sex distribution in accordance with the Federal Statistical Office based on the year 2013 [17] to ensure that the data used was representative of the German population. The research database is considered to have good external validity to the German population in terms of morbidity, mortality, and drug use [18]. The database is fully compliant with all data protection regulations in Germany and has been certified as such.

### Study design and cohort definition

Eligible participants were patients aged 18 years or older. Patient selection was based on the diagnostic codes of the International Classification of Diseases (ICD)-10 [19] and the German procedure classification for the encoding of operations, procedures, and general medical measures [20]. As opioids can be prescribed for indications other than chronic pain, we included only patients with ICD diagnoses suggestive of chronic pain syndromes: diseases of the musculoskeletal system and connective tissue (M00*-M99*), headache syndromes (G43*-G44*, G50.0, G50.1, R10.1), pain unspecified (R52), somatoform pain disorder (F45.4*), other and unspecified polyneuropathies or diabetes mellitus with neurological complications (G62*, or E10.4*-E14.4 plus G63.3).

To reduce the potential for confounding, the cohort was limited to patients without evidence of cancer, palliative, or end-of-life care. Patients were excluded if the cancer diagnosis (C00-C97) was accompanied by at least one claim for radiation therapy (Z51.0; 8-52*) or chemotherapy treatment (Z51.1; 8-541* 8-542* 8-543* 8-544* 8-546*) in the same quarter. Patients requiring palliative care (Z51.5; 8-982*, 8-98e*, 8-98h*) before index date were excluded, as were patients with opioid substitution therapy for opioid addiction (Z51.83) within the study period.

The baseline period was defined as the period prior to the date of the first diagnosis or diagnoses or first opioid or non-opioid treatment initiation as described in the inclusion criteria. The baseline assessment period lasted between January 01, 2012, and first study medication prescription. Patients were included if they had claims in at least three consecutive quarters (quarter = 3 months) between January 1, 2013, and December 31, 2017, with the same diagnosis of chronic pain, thus meeting the criteria for LTOT [21]. Only patients who initiated therapy between 2013 and 2017 and without therapy in 2012 (therapy-naive patients) were included in the study. At least one claim was required between January 1, 2013, and the index treatment. Index treatment was defined as the first claim for a study or control medication between 2013 and 2017.

Patients were identified as receiving a new episode of therapy when they filled a prescription for a study or control medication with no previous fill for medication in that class during the previous year. For inclusion in the study, there was a minimum requirement of three consecutive quarters of study or control medication over the 60-month study period between January 1, 2013, and December 31, 2017. Study medications in the opioid category were all oral or transdermal opioids (buprenorphine, fentanyl, morphine, oxycodone, oxycodone/naloxone, tapentadol, tilidine, tramadol) approved for pain management in Germany. All opioids except tilidine and tramadol can only be accessed by a narcotic prescription and are fully reimbursed by health insurance companies. Control medications were anticonvulsants, antidepressants, dipyrone, and NSAIDs. All treatments were assessed using the Anatomical Therapeutic Chemical codes [22] (Additional file 2, Table 2).

### Matching

New claims for therapy with study medication were matched to new episodes of therapy for the control medication. Mortality rates were expected to be in the order of 100 to 200 per 10,000 person-years, thus limiting the number of covariates for risk adjustment. To avoid overfitting problems, but still be able to adjust for a large number of covariates, a propensity score (PS) matching was applied. PS matching was based on a logistic regression. The choice of covariates was based on Ray et al. [6] (Additional file 3, 3 Table 3).

The 84 covariates included demographic characteristics, diagnoses related to chronic pain, medical procedures including previous surgeries, medication use, diagnoses of mental and somatic diseases, and medical care utilization. Matching on 55 additional binary covariates was planned in the study protocol but could not be used since < 0.1% of patients in one cohort had one characteristic of the covariates. Balance of the propensity score weighting was evaluated using the standardized difference of each covariate (difference in means/percentages over the pooled SD). The standardized differences between the unweighted and propensity-weighted groups were evaluated (Table 1). A standardized difference ≥ 10% is considered a meaningful imbalance between groups [23]. The probability values *p*_*i*_ of the matched patients were allowed to vary by ± 0.2 standard deviation. *C* values > 0.8 are considered to indicate a good classification by the propensity score [24].

**Table 1:** (Selected) characteristics of opioid and non-opioid group before and after matching

Before matching				After matching		
Variable (ATC or ICD 10 or OPS code)	Non-opioid group (*N* = 143.743) %	Opioid-group (*N* = 3415) %	Standardized difference %	Non-opioid group (*N* = 3.223) %	Opioid-group (*N* = 3.223) %	Standardized difference %
Age (years)	Mean 53.2 (SD 16.9)	Mean 69.9 (SD 16.6)	16.7	Mean 66.30 (SD 16.60)	Mean 66.40 (SD.16.77)	0.1
Female gender	51.0	57.0	6.0	56.0	56.0	0
Type 2 diabetes mellitus E11.x	11.0	25.4	38.1	24.9	28.9	9.0
Unspecified diabetes mellitus with kidney complications E14.2	6.3	15.5	29.8	14.9	18.8	10.4
Overweight and obesity E66.x	12.6	19.0	17.6	18.6	17.4	3.1
Disorders of lipoprotein metabolism and other lipidemias E78.x	27.5	39.7	26.0	39.4	41.1	3.5
Major depressive disorder, single episode F32.x	10.9	19.8	24.8	18.5	18.6	0.1
Sleep disorders G47.x	6.7	13.2	22.0	12.7	12.2	1.5
Essential (primary) hypertension I10.x	37.5	61.7	49.8	60.7	61.4	1.5
Heart failure I50.x	3.6	14.3	38.1	13.8	20.9	19.0
Cerebrovascular diseases I60-69	5.3	14.1	29.7	13.6	16.8	9.1
Other chronic obstructive pulmonary disease J44	5.5	14.6	30.8	14.2	16.1	5.2
Dorsalgia M54.x	35.5	54.4	38.7	53.8	42.4	23.0
Number hospitalizations in [t0-365]	19.1	63.2	48.9	59.1	68.9	8.3
Antidiabetics A10	8.0	19.0	32.4	18.7	21.8	7.9
Antithrombotic agents B01	9.0	27.0	48.2	25.9	32.0	13.5
Antiplatelet agents, exkl. heparin B01AC	4.6	13.8	32.1	13.0	19.1	16.8
Acetylsalicylic acid B01AC06	3.7	10.8	27.3	10.0	15.8	17.3
High ceiling diuretics C03C	4.8	22.2	52.7	20.7	28.7	18.7
Beta-adrenoreceptor antagonist C07	19.6	39.3	44.2	38.6	42.3	7.5
Calcium channel blockers C08	9.2	21.5	34.8	21.0	22.3	3.2
Angiotensin-II-antagonists C01C	5.4	10.7	19.5	10.5	12.6	6.8
HMG-CoA-reductase inhibitors C10AA	12.2	24.8	32.9	24.3	28.5	9.5
Corticosteroids, systemic H02Bx	6.4	16.9	33.3	16.7	14.8	4.9
Non-steroidal agents M01A	8.1	41.8	84.6	40.5	32.0	17.8
Analgesics N02	9.4	43.0	82.5	40.9	29.9	23.2
Benzodiazepinderivates N05BA	1.8	6.0	21.5	5.4	5.9	2.4
Antidepressants N06A	1.8	13.0	43.6	10.3	8.7	5.5
Products for obstructive airway diseases R03	10.2	18.3	23.3	17.8	19.0	2.9

*ATC* Anatomical Therapeutic Chemical/Defined Daily Dose Classification, *ICD* International Classification of Diseases, *OPS* Official classification for the encoding of operations, procedures, and general medical measures

The final cohort consisted of 1:1 matched new episodes associated with therapy of the study and the control medication.

### Exposure and follow-up

Patients entered the cohort on the date that the first study or control medication prescription was filled. Exposure time was defined as a maximum of 60 months after the index treatment for each patient. Exposure time ended before the termination of the study period if a patient died, stopped treatment (defined as 1 year without claims for opioids/control medication), changed treatment cohort (from opioids to control medication or vice versa), or was lost to follow-up due to other reasons (e.g., change of insurance), whichever occurred first.

### Endpoints

The primary endpoint was all-cause deaths that occurred during the study follow-up by the date of death in the German claims database. Hospital death was defined as occurring if patients were admitted to a hospital and died during the hospital stay. All other deaths were considered out-of-hospital deaths. In accordance with the German law of data protection, we had no access to death certificates.

### Statistical analysis

Opioid dosage was calculated based on morphine equivalent (MEQ) values as time-varying covariates, with annual recalculations during follow-up. For MEQ calculation, the ATC classification with defined daily doses (DDD) was adapted and multiplied with the equivalent factor to calculate the oral morphine equivalent [22]. The average daily MEQ/day dispensed was then calculated for 365 days’ exposure by summing the morphine equivalents for the prescriptions dispensed for the 365-day period and dividing this number by 365. Opioids prescribed during hospital stays during the study period were not included in the calculation of MEQ/day because these data were not available.

The statistical analysis compared the adjusted risk of all-cause death during follow-up for patients in the opioid cohort with those in the control medication cohort. We calculated mortality for each cohort and plotted Kaplan-Meier mortality curves. We compared mortality in the opioid cohort with the control medication cohort using multivariate Cox proportional hazard models. Relative risk was estimated with the hazard ratio (HR), calculated from the Cox regression models. The proportional hazard assumption was checked visually by visual inspections of the weighted residuals versus time*.* We found no evidence that this assumption was violated.

The model included age, gender, quarter of index treatment, estimated propensity score, Charlson Comorbidity Index (CCI) based on the definition by Quan et al. [25] (Additional file 4, Table 4), study opioid cohort, and treatment duration as covariates. Treatment duration was defined as cumulative dispensed days of therapy on the day a study drug prescription was filled. A sensitivity analysis excluded all patients with at least one C00–C96 diagnosis before index date. We considered a HR ≥ 1.57 to be clinically relevant assuming an exponentially distributed survival time [26].

Based on the findings of Zeng et al. [7] (all-cause mortality of 4.0% in the tramadol and 2.5% in the NSAID group after the first year of prescription), and assuming a constant mortality rate per year in our five study periods, 405 participants per group would provide at least 80% power to show statistical significance between opioid- and non-opioid groups for all-cause mortality at an overall 2-sided significance level of < 0.05.

Subgroup analyses that assessed special populations were performed: pain, not specified (R52*); persistent somatoform pain disorder (F45.4*); osteoarthritis (M 15*-M19*), low back pain (M54*), diabetic polyneuropathy (E10.4*-E14.4 plus G63.3). All-cause mortality between the subgroups was compared by log rank test and is presented as risk difference. In addition, stratified analyses according to MEQ/day were performed. The cut-point for high-dose opioid therapy was set at ≥ 100 mg MEQ/day.

The GenMatch algorithm was run with R Project for Statistical Computing. The *pvals* function was used in the *fit.func* argument. The propensity scores were estimated using a multivariate logistic regression (logit model). All other analyses were performed with SAS version 9.4. All *p* values were 2-sided.

### Ethical approval

This study was a retrospective database analysis based on fully anonymized claims data. Claims data were recorded for accounting purposes and not for clinical research. No electronic medical records or other clinical parameters were used. As a result, no ethical approval or consent from an ethics committee or review board was required for this study.

## Results

**Fig. 1:**
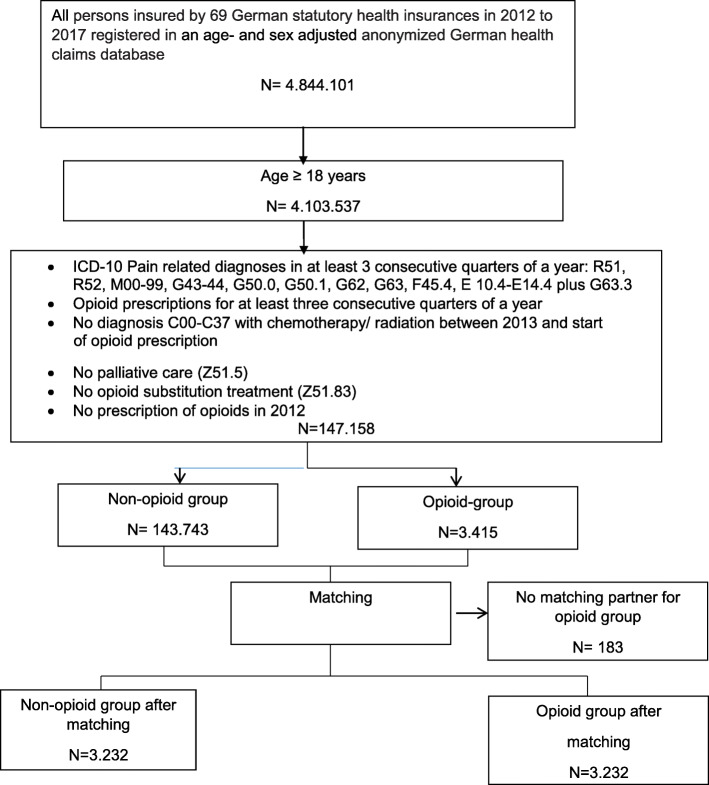
PRISMA flow diagram

The groups differed with standardized differences exceeding 10% for most study covariates before matching: Participants in the opioid group were older and had a higher prevalence of somatic and psychiatric comorbidities treated with drug therapies and more previous hospital stays. After matching, the cohort included 3232 new episodes of prescriptions for opioids and an equal number of control medication episodes (see Fig. 1). There was a standardized difference ≥ 10% for three comorbidities in favor of opioids and one comorbidity in favor of non-opioids. There was a standardized difference ≥ 10% for nine medications: seven were more frequent in the opioid group and two more frequent in the non-opioid group (Table 1, Additional files 5 and 6, Tables 5 and 6). *C* score of the propensity score was 0.84.

The most frequent chronic pain syndromes in the opioid group were low back pain (22.6%), osteoarthritis (22.2%), pain not specified (9.7%), somatoform pain disorder (6.5%), and diabetic polyneuropathy (2.4%).

LTOT patients had 554 deaths during 10,435 person-years (531 per 10,000 person-years), whereas there were 340 deaths during 11,342 person-years (300 per 10,000 person-years) in the control group. The RD was 148 (95% CI 99, 198) excess deaths per 10,000 person-years. The adjusted HR for all-cause death during follow-up was 1.58 (95% CI 1.38, 1.82). The number needed to treat for an additional death was 16 (95% CI 11, 25).

In addition, all-cause mortality was predicted by male gender, increasing age, and duration of therapy (Table 2). The magnitude of HR for opioid therapy, but not for other predictors met the criterion of a relevant difference.

**Table 2:** Predictors of all-cause mortality in the study sample (*N* = 6464)

Predictor	Adjusted HR (95% CI); *p* value
Gender	
Male	1.29 (1.12–1.48); 0.0003
Female	Referent
Age (per year)	1.09 (1.08–1.10); < 0.0001
Long-term opioid therapy	1.58 (1.38–1.82); < 0.0001
Non-opioid therapy	Referent
Duration of drug therapy (per month)	0.996 (0.995–0.996); < 0.0001
Comorbidity index	1.19 (1.16–1.23); < 0.0001
Estimated propensity score	1.32 (0.96–1.81); 0.08
Index quarter	0.998 (0.998–0.999); < 0.0001

*CI* confidence interval

The sensitivity analysis of all-cause mortality yielded an adjusted HR 1.35 (95% CI 1.14, 1.58) (Additional file 7, Table 7).

The elevated risk of death for LTOT was confined to the out-of-hospital deaths: LTOT patients had 288 out-of-hospital deaths during 10,435 person-years (276 per 10,000 person-years), whereas there were 110 deaths during 11,342 person-years (97 per 10,000 person-years) in the control group. Adjusted HR was 2.29 (95% CI 1.86, 2.83). LTOT patients had 268 in-hospital deaths during 10,435 person-years (97 per 10,000 person-years), whereas there were 229 deaths during 11,342 person-years (202 per 10,000 person-years) in the control group. Adjusted HR was 1.16 (95% CI 0.96, 1.42).

Subgroup analyses that assessed populations of particular interest found similar results for all-cause mortality according to the primary analysis, except for diabetic polyneuropathy (Table 3).

**Table 3:** Subgroup analyses

Subgroup	***N*** (opioid)	***N*** (non-opioid)	Death (opioid)	Death (non-opioid)	Risk difference (95% confidence interval)	Person-years follow-up (opioid)	Person-years follow-up (non-opioid)	Incidence death per 10,000 person-years (opioid)	Incidence death per 10,000 person-years (non-opioid)
Osteoarthritis	718	724	151	92	0.06 (0.03–0.09)	2250.2	2477.1	671.0	371.4
Low back pain	667	722	75	40	0.08 (0.04–0.12)	2162.0	2535.5	346.9	157.8
Pain, unspecified	651	313	141	33	0.11 (0.08–0.16)	795.5	326.8	795.5	326.8
Diabetic polyneuropathy	76	73	16	22	− 0.09 (− 0.23–0.06)	187.6	197.0	852.7	1116.7

Adjusted HR for all-cause death was 1.64 (955 CI 1.43, 1.89) for MEQ < 100 mg/day and 1.59 (95% CI 1.38, 1.82) for MEQ ≥ 100 mg/day (Additional files 8 and 9, Tables 8 and 9).

## Discussion

This study has identified that patients with LTOT for CNCP had a risk of all-cause mortality 1.58 times greater than for matched patients initiating an analgesic treatment with anticonvulsants, antidepressants, dipyrone, or NSAIDs. This finding corresponds to 148 excess deaths per 10,000 person-years of therapy. This difference was explained by a 2.57 times greater risk of out-of-hospital deaths.

The adjusted HRs for all-cause mortality were comparable between the studies with propensity-matched analysis: HR was 1.64 in Ray et al. [6], 1.71 in Zeng et al. [7], and 1.58 in this current study.

Our study confirms the findings of Ray et al. [6] that all-cause mortality was restricted to out-of-hospital deaths. Out-of-hospital deaths likely better reflect adverse medication effects than in-hospital deaths, which might be influenced by hospital admission for more severe disease requiring hospital treatment [27]. In addition, adverse respiratory effects of opioids might be better managed in hospital than in ambulatory care.

The propensity-matched studies available cannot confirm the recommended threshold dosages for opioids to treat CNCP of current guidelines [9, 10]: The confidence intervals of HR for all-cause mortality in the study of Ray overlapped: HR was 1.54 (1.01, 2.34) for low dose (≤ 60 mg MEQ) and was 1.94 (1.40, 2.70) for high doses (> 60 mg MEQ/day) [6] as did the confidence intervals in our study.

This current study was designed to reduce confounding by factors associated with starting LTOT for CNCP. The cohort excluded patients with evidence of cancer, palliative care, and opioid substitution for opioid addiction. Inclusion was restricted to those initiating therapy with study medications.

It is important to consider whether the elevated risk for LTOT was due to confounding by indication. Patients with initial prescriptions of opioids were older, had more comorbidities, and received more drug treatment than non-opioid patients before matching. To control for confounding by indication, patients in the two study groups were matched according to potential confounders, including somatic and psychiatric comorbidities and healthcare use. Although our propensity score matching model indicated a good classification, residual confounding cannot be fully excluded. The opioid group had a higher prevalence of heart failure and a higher use of anti-thrombotic and antiplatelet agents and psycholeptics.

Opioids as well as anticonvulsants, antidepressants, and NDAIDs are frequently used for the most frequent recorded diagnoses in our cohort, namely osteoarthritis and low back pain [28]. In contrast to other countries, dipyrone is frequently used in Germany [29]. Material confounding by indication seems unlikely because the findings restricted to patients with a diagnosis of low back pain and osteoarthritis remained essentially unchanged.

### Strengths and limitations of this study

This study was based on a large patient sample of German statutory health insurances which is representative of the German population in terms of age and gender. The study was conducted in a healthcare system with free access to multicomponent pain treatments that are fully reimbursed by health insurance companies and within a strict framework for secure prescription procedures for opioids. There is at this time no signal for an opioid epidemic in Germany [15] which might confound data on opioid-associated mortality.

The major limitations are as follows:

- Due to the administrative nature of the database, the possibility of misclassification and miscoding of data exists.
- There was no clinical patient information, and we did not know whether the dispensed drugs were used immediately, saved for later use, diverted, or not used at all. The assumption that all drugs were used at a time close to dispensing likely causes an overestimation of drug use.
- Our primary data did not provide demographic information such as education level, income, or data on lifestyle (e.g., smoking, physical activity). Analyses in this study were unadjusted for these potential covariates (unmeasured confounding).
- We might have underestimated all-cause mortality associated with opioid therapy, because we analyzed only patients with consecutive prescriptions > 270 days. Therefore, we did not capture patients who died in the first months of opioid treatment.
- Due to German laws of data protection, causes of deaths could not be ascertained. Therefore, we could not test the increased risk of cardiovascular deaths in the opioid group as reported by Ray et al. [6]. LTOT is associated with changes in sleep architecture and an increased risk of respiratory depression during sleep [30]. Sleep-disordered breathing caused by opioids in moderate dosages alone or in combination with tranquilizers might increase the incidence of nocturnal arrhythmias and myocardial ischemia [6, 31]. In addition, some opioids such as tramadol and oxycodone in high doses may develop long QT interval and ventricular tachycardia [32]. These cardiorespiratory changes are likely more frequently detected and treated during a hospital stay than in an outpatient setting.
  The reliability and accuracy of information provided on German death certificate is poor [33]. Therefore, we do not believe that analysis of causation of death as assessed by German death certificates would have contributed additional reliable information.
- The contract with the health insurance companies excluded other analyses than the ones defined in the study protocol. Therefore, we were not able to conduct a post hoc sensitivity analysis excluding participants with heart failure and use of anti-thrombotic and antiplatelet agents and of psycholeptics to adjust for the potential higher risk of death in the opioid group.

## Conclusions

When discussing treatment options for patients with CNCP, the relevant risk of increased all-cause mortality associated with opioids should be included to the numerous known harms that associate with this category of drugs. Nevertheless, for some patients, the therapeutic benefits from LTOT may outweigh the relevant increase in mortality risk, especially if the alternative drug treatments are less effective, poorly tolerated, or contraindicated. In North American and European guidelines, opioids remain one drug treatment option in carefully selected and monitored patients with CNCP [9, 10].

## Supplementary information

**Additional file 1: Table S1.** STROBE CHECKLIST.

**Additional file 2: Table S2.** Anatomical Therapeutic Chemical codes.

**Additional file 3: Table S3.** Covariates of the study of Ray [6].

**Additional file 4: Table S4.** Creation of Charlson Comorbidity Index based on health insurance claims data.

**Additional file 5: Table S5.** Distribution of all covariates before and after matching.

**Additional file 6: Table S6.** Definition of low dose and high dose anticonvulsants and antidepressants.

**Additional file 7: Table S7.** Sensitivity analysis of predictors of all-cause mortality in the study sample (*N* = 2813 in non-opioid and *N* = 2757 in opioid group).

**Additional file 8: Table S8.** Predictors of all-cause mortality in patients with < 100 MEQ/.

**Additional file 9: Table S9.** Predictors of all-cause mortality in patients with ≥100 MEQ/d.

## Data Availability

The data that support the findings of this study are not publically available as they are owned by the German statutory health insurances. The dataset from this study is held securely in coded form at the Institute for Applied Health Research (*InGeF*)*.* While data sharing agreements prohibit InGeF from publicly releasing a minimal deidentified dataset, access can be granted to those who meet pre-specified criteria for confidential access through LinkCare. More information on how to access this data is available at https://www.link-care.de/deutsch/leistungen/versorgungsforschung/. All analytical requests must be approved by InGef.

## Abbreviations

CCI: Charlson Comorbidity Index
CI: Confidence interval
CNCP: Chronic non-cancer pain
DDD: Defined daily doses
HR: Hazard ratio
ICD: International Classification of Diseases
MEQ: Morphine equivalent
NSAIDs: Non-steroidal anti-inflammatory drugs
RD: Risk difference
