# Supplementary material for: All-cause mortality in patients with long-term opioid therapy compared with non-opioid analgesics for chronic non-cancer pain: a database study
Source: BMC Med. 2020 Jul 15;18:162. doi: 10.1186/s12916-020-01644-4 (PMC7362543; doi:10.1186/s12916-020-01644-4)
Supplement: Supplementary file 1 — Additional file 1: Table S1. STROBE CHECKLIST. [file 12916_2020_1644_MOESM1_ESM.docx]

**Additional file 1, Table 1: Strobe Checklist**

|  | | Item No | Recommendation Page |
| --- | --- | --- | --- |
| **Title and abstract** | | 1 | Indicate the study’s design with a commonly used  term in the title or the abstract 1 |
|  |  |  | Provide in the abstract an informative and  balanced summary of what was done and what was 3  found |
| Introduction | | | |
| Background/rationale | | 2 | Explain the scientific background and rationale  for the investigation being reported 4 |
| Objectives | | 3 | State specific objectives, including any prespecified  hypotheses |
| Methods | | | |
| Study design | | 4 | Present key elements of study design early in the 6  paper |
| Setting | | 5 | Describe the setting, locations, and relevant dates,  including periods of recruitment, exposure, follow-up, 6-8  and data collection |
| Participants | | 6 | *Cohort study*—Give the eligibility criteria, and the  sources and methods of selection of participants.  Describe methods of follow-up |
|  |  |  | (*b*) *Cohort study*—For matched studies, give matching  criteria and number of exposed and unexposed 6-8 |
| Variables | | 7 | Clearly define all outcomes, exposures, predictors,  potential confounders, and effect modifiers. Give 6-7  diagnostic criteria, if applicable |
| Data sources/ measurement | | 8* | For each variable of interest, give sources of data  and details of methods of assessment (measurement). 6-8  Describe comparability of assessment methods if there  is  more than one group |
| Bias | | 9 | Describe any efforts to address potential sources of bias 10 |
| Study size | | 10 | Explain how the study size was arrived at 11 |
| Quantitative variables | | 11 | Explain how quantitative variables were handled in the  analyses. If applicable, describe which groupings were  chosen and why |
| Statistical methods | | 12 | Describe all statistical methods, including those 10-11  Sed to control for confounding |
|  |  |  | Describe any methods used to examine subgroups and interactions 10-11 |
|  |  |  | Explain how missing data were addressed |
|  |  |  | *Cohort study*—If applicable, explain how loss to follow-up N/A  was addressed |
|  |  |  | Describe any sensitivity analyses 11 |
| Results | | | |
| Participants | 13* | 1. Report numbers of individuals at each stage of study— 2. eg numbers potentially eligible, examined for eligibility, 3. confirmed eligible, included in the study, completing 4. follow-up, and analyzed figure 1 | |
|  |  | (b) Give reasons for non-participation at each stage N/A | |
|  |  | © Consider use of a flow diagram figure 1 | |
| Descriptive data | 14* | a)Give characteristics of study participants (eg demographic, table 1  clinical, social) and information on exposures and potential confounders | |
|  |  | (b) Indicate number of participants with missing data for each variable of interest N/A | |
|  |  | (c) *Cohort study*—Summarize follow-up time (eg, average and total amount) | |
| Outcome data | 15* | *Cohort study*—Report numbers of outcome events or summary  measures over time 8 | |
| Main results | 16 | (*a*) Give unadjusted estimates and, if applicable, confounder-  adjusted estimates and their precision (eg, 95% confidence interval). Make clear which confounders were adjusted for and why they were included  12-13; Table 3; Table S7 | |
|  |  | (*b*) Report category boundaries when continuous variables were categorized  N/A | |
|  |  | (*c*) If relevant, consider translating estimates of relative risk into absolute risk for a meaningful time period table 3 | |
| Other analyses | 17 | Report other analyses done—eg analyses of subgroups and interactions, and sensitivity analyses 13 | |
| Discussion | | | |
| Key results | 18 | Summarize key results with reference to study objectives 13-14 | |
| Limitations | 19 | Discuss limitations of the study, taking into account sources of  potential bias or imprecision. Discuss both direction and magnitude  of any potential bias 14-15 | |
| Interpretation | 20 | Give a cautious overall interpretation of results considering objectives, limitations, multiplicity of analyses, results from similar studies, and  other relevant evidence 14 | |
| Generalisability | 21 | Discuss the generalisability (external validity) of the study results 14-17 | |
| Other information | | | |
| Funding | 22 | Give the source of funding and the role of the funders for the present study and, if applicable, for the original study on which the present  article is based 2 | |
|  |  |  | |
