# Supplementary material for: All-cause mortality in patients with long-term opioid therapy compared with non-opioid analgesics for chronic non-cancer pain: a database study
Source: BMC Med. 2020 Jul 15;18:162. doi: 10.1186/s12916-020-01644-4 (PMC7362543; doi:10.1186/s12916-020-01644-4)
Supplement: Supplementary file 2 — Additional file 2: Table S2. Anatomical Therapeutic Chemical codes. [file 12916_2020_1644_MOESM2_ESM.docx]

Additional file 2, Table 2: List of ATC codes of study opioid and non-opioids

| **Name** | **ATC Code** |
| --- | --- |
| **Study opioids** | |
| Morphine | N02AA01 |
| Oxycodone | N02AA05 |
| Fentanyl | N02AB03 |
| Buprenorphine | N02AE01 |
| Tramadol | N02AX02 |
| Tilidine | N02AX01 |
| Tapentadol | N02AX06 |
| **Study non-opioids (anticonvulsants)** | |
| Gabapentin | N03AX12 |
| Pregabalin | N03AX16 |
| Carbamazepine | N03AF01 |
| **Study non-opioids (antidepressants)** | |
| Non-selective monoamine reuptake inhibitors | N06AA* |
| Selective serotonin reuptake inhibitors | N06AB* |
| Monoamine oxidase inhibitors, non-selective | N06AF* |
| Monoamine oxidase A inhibitors | N06AG* |
| Other antidepressants | N06AX* |
| **Study non-opioids (Nonsteroidal anti-inflammatory drugs)** | |
| Butylpyrazolidines | M01AA* |
| Acetic acid derivatives and related substances | M01AB* |
| Oxicams | M01AC* |
| Propionic acid derivatives | M01AE* |
| Fenamates | M01AG* |
| Coxibs | M01AH* |
| Other antiinflammatory and antirheumatic agents, non-steroids | M01AX* |
| **Study non-opioids (others)** | |
| Dipyrone | N02BB02 |
