# Supplementary material for: All-cause mortality in patients with long-term opioid therapy compared with non-opioid analgesics for chronic non-cancer pain: a database study
Source: BMC Med. 2020 Jul 15;18:162. doi: 10.1186/s12916-020-01644-4 (PMC7362543; doi:10.1186/s12916-020-01644-4)
Supplement: Supplementary file 3 — Additional file 3: Table S3. Covariates of the study of Ray [6]. [file 12916_2020_1644_MOESM3_ESM.docx]

**Additional file 3, Table 3: Comparison of matching variables of Ray [6] and this study**

|  |  |  |
| --- | --- | --- |
| **Variables of Ray** | **Operationalisation (if possible)** | **Matching variable in this study** |
| Sex (female) | Indicated sex in the social insurance register | Yes |
| White race | Not possible | No; not included in database |
| Age at baseline, in years | Based on birth date as indicated in the social insurance register | Yes |
| Standard Metropolitan Statistical Area | Based on region (‘Bundesland’) as indicated in the social insurance register | No; not included in database |
| Medicaid enrollment disabled | Not possible | No; not included in database |
| Year: 2012 | Number of eligible patients in the social insurance register | Yes |
| Year: 2013 | Number of eligible patients in the social insurance register | Yes |
| Year: 2014 | Number of eligible patients in the social insurance register | Yes |
| Year: 2015 | Number of eligible patients in the social insurance register | Yes |
| Year: 2016 | Number of eligible patients in the social insurance register | Yes |
| Back pain past 90 days | ICD-10 Code M54* coded in last three quarters as inpatient or ensured outpatient diagnosis | Yes |
| Other musculoskeletal pain past 90 days | ICD-10 Code R52* coded in last three quarters  as inpatient or ensured outpatient diagnosis | Yes |
| Abdominal pain past 90 days | ICD-10 Code R10* coded in last three quarters as inpatient or ensured outpatient diagnosis | Yes |
| Headache past 90 days | ICD-10 Code R51, G43*, G44*, G50.0, G50.1coded in last three quarters as inpatient or ensured outpatient diagnosis | No |
| Other neurologic pain past 90 days | ICD-10 Code E10.4-E14.4 AND G63.3 coded in last three quarters as inpatient or ensured outpatient diagnosis | Yes |
| Pesistent back pain | M54,- | Yes |
| Pesistent musculoskeletal pain | M79.6 | Yes |
| Pesistent abdominal pain | R10.- | Yes |
| Pesistent headache | R51.- | Yes |
| Pesistent neurologic pain | M79.2 | Yes |
| Rheumatoid arthritis/ other inflammatory arthropathy | ICD-10 Code M05-M14 as inpatient or ensured outpatient diagnosis | Yes |
| Current opioid, ≤60mg morphine equivalents | N02AA01, N02AA03, N02AA05, N02AX02, N02AX51, N02AE01, N02AB02, N02AX06, N07BC02 N02AB03 , N02AC03 | No; only opioid-naive patients  Included |
| Current opioid, 60-120mg morphine equivalents | N02AA01, N02AA03, N02AA05, N02AX02, N02AX51, N02AE01, N02AB02, N02AX06, N07BC02 N02AB03 , N02AC03 | No; only opioid-naive patients  Included |
| Current opioid, >120mg morphine equivalents | N02AA01, N02AA03, N02AA05, N02AX02, N02AX51, N02AE01, N02AB02, N02AX06, N07BC02 N02AB03 , N02AC03 | No; only opioid-naive patients  included |
| Opioid, any | N02AA01, N02AA03, N02AA05, N02AX02, N02AX51, N02AE01, N02AB02, N02AX06, N07BC02 N02AB03 , N02AC03 | No; only opioid-naive patients  included |
| Opioid, 91-180 days use prior year | N02AA01, N02AA03, N02AA05, N02AX02, N02AX51, N02AE01, N02AB02, N02AX06, N07BC02 N02AB03 , N02AC03 | No; only opioid-naive patients  Included |
| Opioid, 181-270 days use prior year | N02AA01, N02AA03, N02AA05, N02AX02, N02AX51, N02AE01, N02AB02, N02AX06, N07BC02 N02AB03 , N02AC03 | No; only opioid-naive patients  Included |
| Opioid, >270 days use prior year | N02AA01, N02AA03, N02AA05, N02AX02, N02AX51, N02AE01, N02AB02, N02AX06, N07BC02 N02AB03 , N02AC03 | No; only opioid-naive patients  Included |
| Skeletal muscle relaxant, any | M03A* | Yes |
| Skeletal muscle relaxant, >270 days use prior year | M03A* | Yes |
| Non-steroidal antiinflammatory drug, any | M01A* | Yes |
| Non-steroidal antiinflammatory drug, >180 days use prior year | M01A* | Yes |
| Corticosteroid, any | H02*, R01AD* | Yes |
| DMARD (Disease-modifying antirheumatic drug), any | A07EC, L01BA, L04AA, L04AX, M01C, P01BA | Yes |
| Other analgesic, any | other N02* | Yes |
| Benzodiazepine, any | N05BA* | Yes |
| Benzodiazepine, current, <5mg diazepam | N05BA* | Yes |
| Benzodiazepine, current, >5mg | N05BA* | Yes |
| Benzodiazepine, 90-180 days use prior year | N05BA* | Yes |
| Benzodiazepine, 181-270 days use prior year | N05BA* | Yes |
| Benzodiazepine, >270 days use prior year | N05BA* | Yes |
| Antipsychotic, any | N05A* | Yes |
| Zopiclon | N05CF01 | No, due to few patients |
| Zolpidem | N05CF02 | No, due to few patients |
| Zaleplon | N05CF03 | No, due to few patients |
| SSRI/SNRI | N06AB* | Yes |
| Trazodone | N06AX05 | Yes |
| Other antidepressant | Other N06A* | Yes |
| cannabinoid drugs | N02BG10, A04AD10, A04AD11, V90RA, V90RB | Yes |
| Other GABA agonist |  | Yes |
| Hydroxyzine | N05BB01 | Yes |
| Other anxiolytic | Other N05* | Yes |
| Schizophrenia or other psychosis | ICD-10 Code F20-F29 as inpatient or ensured outpatient diagnosis | Yes |
| Bipolar | ICD-10 CodF31.- as inpatient or ensured outpatient diagnosis | No, due to few patients |
| Depression | ICD-10 Code F32.-, F33.- as inpatient or ensured outpatient diagnosis | Yes |
| Other affective disorder | ICD-10 Code F38.- as inpatient or ensured outpatient diagnosis | Yes |
| Sleep disorder | ICD-10 Code F51.-, G47.- as inpatient or ensured outpatient diagnosis | Yes |
| Panic disorder | ICD-10 Code F40.01, F40.02, F41.0 as inpatient or ensured outpatient diagnosis | Yes |
| Anxiety disorder | ICD-10 Code F41.- as inpatient or ensured outpatient diagnosis | Yes |
| ACE inhibitor | CA09A, C09B | No, due to few patients |
| Angiotensin receptor blocker | C09CA | Yes |
| Anticoagulant | B01* | Yes |
| Aspirin | N02BA01, B01AC06 | Yes |
| Beta blocker | C07* | Yes |
| Calcium channel blocker | C08* | Yes |
| Digoxin | C01AA05 | Yes |
| Loop diuretic | C03C | Yes |
| Other diuretic | Other C03 | Yes |
| Insulin | A10 | Yes |
| Oral hypoglycemic | A10X | Yes |
| Statin | C10AA | Yes |
| Fibrate | C10AB | Yes |
| Nitrate | C01DA | Yes |
| Platelet inhibitor | B01AC | Yes |
| New cardiovascular medication start |  | Yes |
| Serious coronary heart disease | I20* - I25* | Yes |
| Cardiac valve disorder | ICD-10 Code I34.-, I35.-, I36.-, I37.-, I38.- 39.-* as inpatient or ensured outpatient diagnosis | Yes |
| Arrhythmia | ICD-10 Code I49.-as inpatient or ensured outpatient diagnosis | Yes |
| Congestive heart failure | ICD-10 Code I50* as inpatient or ensured outpatient diagnosis | Yes |
| Cerebrovascular disorder | I60*-I69* ICD-10 Code as inpatient or ensured outpatient diagnosis | Yes |
| Peripheral vascular disease | ICD-10 Code I73*, I74.2, I74.3, I74.4, I71.3, I71.5, I71.4, I71.6 as inpatient or ensured outpatient diagnosis | Yes |
| Diabetes | ICD-10 Code E10-E14 as inpatient or ensured outpatient diagnosis | Yes |
| Hypertension | ICD-10 Code I10-I15 coded as inpatient or ensured outpatient diagnosis | Yes |
| Hyperlipidemia | ICD-10 Code E78.- as inpatient or ensured outpatient diagnosis | Yes |
| Other cardiovascular diseases | ICD-10 Code I95.-I99.- as inpatient or ensured outpatient diagnosis | Yes |
| Obesity | ICD-10 Code E66.- as inpatient or ensured outpatient diagnosis | Yes |
| Smoking | Not possible | No; variable not included in databasse |
| Diabetes complications | Fourth number from 0 until 8 of ICD-10 Code E10-E14 as inpatient or ensured outpatient diagnosis | Yes |
| Diabetes poor control | Not possible | No; variable not included in databasse |
| Diabetes hospitalization | ICD-10 Code E10-E14 as inpatient admission diagnosis in last three quarters | Yes |
| New cardiovascular diagnosis | ICD-10 Code I34.-, I35.-, I36.-, I37.-, I38.- 39.-*, I49.-, I50*, I60*-I69*, I73*, I74.2, I74.3, I74.4, I71.3, I71.5, I71.4, I71.6, I95.-I99.- as new inpatient or ensured outpatient diagnosis | Yes |
| Unintentional fall | ICD-10 Code V01.-Y84.- as inpatient or ensured outpatient diagnosis | Yes |
| Wheelchair or walker | Not possible | No; variable not included in databasse |
| Incontinence | ICD-10 Code R32.-, N39.4, or N39.3 as inpatient or ensured outpatient diagnosis | Yes |
| Other frailty | ICD-10 Code R54.- as inpatient or ensured outpatient diagnosis | Yes |
| Home health care | Not possible | No; variable not included in databasse |
| Beta agonist | R03AA, R02BB, R03AC, R03CB, R03CC | Yes |
| Other bronchodilator | Other R03 | Yes |
| COPD | ICD-10 Code J44.- as inpatient or ensured outpatient diagnosis | Yes |
| Asthma | ICD-10 Code J45.- as inpatient or ensured outpatient diagnosis | Yes |
| Home oxygen | V03AN01 | No, due to few patients |
| Anticonvulsant | N03A | Yes |
| Seizure disorder | G40* | Yes |
| Hospitalization in [t0-365] |  | Yes |
| ED past 30 days | hospitalisation in last 1 quarter with emergency admission reason 07 | Yes |
| Cardiovascular outpatient visit past year | outpatient visit of cardiologist in the last 4 quarters | No |
| Outpatient visits past year: 6-20 | number of outpatient visits per patient and year between 6 and 20 in the last 4 quarters | No |
| Outpatient visits past year: 21-60 | number of outpatient visits per patient and year between 21 and 60 in the last 4 quarters | No |
| Outpatient visits past year: >60 | number of outpatient visits per patient and year more than 60 in the last 4 quarters | No |
| Injury inpatient stay | Not possible | No; variable not included in databasse |
| Injury ED visit | Not possible | No; variable not included in databasse |
| Injury outpatient visit | Not possible | No; variable not included in databasse |
| Overdose inpatient stay/ED visit | T36-T50 | No, due to few patients |
