# Supplementary material for: All-cause mortality in patients with long-term opioid therapy compared with non-opioid analgesics for chronic non-cancer pain: a database study
Source: BMC Med. 2020 Jul 15;18:162. doi: 10.1186/s12916-020-01644-4 (PMC7362543; doi:10.1186/s12916-020-01644-4)
Supplement: Supplementary file 4 — Additional file 4: Table S4. Creation of Charlson Comorbidity Index based on health insurance claims data. [file 12916_2020_1644_MOESM4_ESM.docx]

Additional file 4, Table 4: Creation of Charlson Comorbidity Index [24] based on health insurance claims data

| **Condition** | **Scale** | **Points** | **ICD-10-GM Code** |
| --- | --- | --- | --- |
| Myocardial infarction | No | 0 |  |
|  | yes | 1 | I21*-I22*, I25.2 |
| Congestive Heart Failure | No | 0 |  |
|  | yes | 1 | I50* |
| Peripheral vascular disease | No | 0 |  |
|  | yes | 1 | I73*, I74.2, I74.3, I74.4, I71.3, I71.5, I71.4, I71.6 |
| Cerebrovascular accidents | No | 0 |  |
|  | yes | 1 | I60*-I69* |
| Dementia | No | 0 |  |
|  | yes | 1 | F00*-F03*, F05.1 |
| Chronic obstructive pulmonary disease | No | 0 |  |
|  | yes | 1 | J44* |
| Rheumatic disease | No | 0 |  |
|  | yes | 1 | M05*, M06*, M31.5, M32–M34, M35.3, M45*, |
| Peptic ulcer disease | No | 0 |  |
|  | yes | 1 | K25*, K26*, K27*, K28* |
| Liver disease | No | 0 |  |
|  | Mild | 1 | K70.1-K70.3, K70.9, K71-K71.0, K71.2-K71.8, K73*, K74.0, K74.2, K74.6, K74.3, K74.4, K74.5 |
|  | Severe | 3 | K70.4, K71.1, K72*, K76.3 |
| Diabetes mellitus | No | 0 |  |
|  | Un-complicated | 1 | E10.9*, E11.9*, E13.9*, E14.9*, E10.1*, E11.1*, E13.1*, E14.1* |
|  | Complicated / end organ damage | 2 | E10.2*, E11.2*, E13.2*, E14.2* E10.3*, E11.3*, E13.3*, E14.3* E10.4*, E11.4*, E13.4*, E14.4*, E10.5*, E11.5*,  E13.5*, E14.5* |
| Hemiplegia | No | 0 |  |
|  | yes | 2 | G81* |
| Kidney diseases | No | 0 |  |
|  | yes | 2 | N17*-N19* |
| Cancer | No | 0 |  |
|  | Solid | 2 | C00*-C76*, C80*, C81*, C82*, C83*, C84*, C88.3, C88.7, C88.9, C96*, C90.1, C91*-C95*, C81*, C82*, C83*, C84*, C85*, C86*, C88* |
|  | Metastatic | 6 | C77*, C78*, C79*, C80* |
| AIDS / HIV | No | 0 |  |
|  | yes | 6 | B20* - B24* |
