# Supplementary material for: All-cause mortality in patients with long-term opioid therapy compared with non-opioid analgesics for chronic non-cancer pain: a database study
Source: BMC Med. 2020 Jul 15;18:162. doi: 10.1186/s12916-020-01644-4 (PMC7362543; doi:10.1186/s12916-020-01644-4)
Supplement: Supplementary file 5 — Additional file 5: Table S5. Distribution of all covariates before and after matching. [file 12916_2020_1644_MOESM5_ESM.docx]

| **Additional file 5, Table 5:** **Distribution of all covariates before and after matching**   \| **Before matching** \| \| \| \| **After matching** \| \| \| \| --- \| --- \| --- \| --- \| --- \| --- \| --- \| \| **Variable (ATC or ICD 10 or OPS-code)** \| **Non-opioid group (N=143.743) %** \| **Opioid-group (N=3415) %** \| **Standardized difference %** \| **Non-opioid group (N=3.223) %** \| **Opioid-group (N=3.223) %** \| **Standardized difference %** \| \| \| Type 1 diabetes mellitus E10.x \| 1.5 \| 4.1 \| 15,  .9 \| 3.9 \| 5.1 \| 5.8 \| \| \| Type 2 diabetes mellitus E11.x \| 11.0 \| 25.4 \| 38.1 \| 24.9 \| 28.9 \| 9.0 \| \| \| Type 2 diabetes mellitus with kidney complications E11.2 \| 0.6 \| 2.2 \| 13.5 \| 2.0 \| 3.2 \| 7.2 \| \| \| Type 2 diabetes mellitus with ophthalmic complications E11.3 \| 0.9 \| 2.1 \| 10.1 \| 2.2 \| 3.0 \| 5.2 \| \| \| Type 2 diabetes mellitus with neurological complications E11.4 \| 1.5 \| 4.6 \| 18.0 \| 4.3 \| 6.2 \| 8.4 \| \| \| Type 2 diabetes mellitus with multiple complications E11.7 \| 1.1 \| 3.7 \| 16.8 \| 3.7 \| 5.5 \| 8.8 \| \| \| Type 2 diabetes mellitus with unspecified complications E11.8 \| 0.8 \| 1.9 \| 10.1 \| 1.7 \| 2.6 \| 6.2 \| \| \| Unspecified diabetes mellitus with kidney complications E14.2 \| 6.3 \| 15.5% \| 29.8 \| 14.9 \| 18.8 \| 10.4 \| \| \| Unspecified diabetes mellitus with ophthalmic complications E14.3 \| 1.0 \| 2.3 \| 10.2 \| 2.2 \| 3.0 \| 8.2 \| \| \| Unspecified diabetes mellitus with neurology complications E14.4 \| 0.6 \| 2.3 \| 14.3 \| 2.1 \| 3.1 \| 6.0 \| \| \| Overweight and obesity E66.x \| 12.6 \| 19.0 \| 17.6 \| 18.6 \| 17.4 \| 3.1 \| \| \| Disorders of lipoprotein metabolism and other lipidemias E78.x \| 27.5 \| 39.7 \| 26.0 \| 39.4 \| 41.1 \| 3.5 \| \| \| Schizophrenia F2x \| 0.6 \| 1.1 \| 5.3 \| 1.1 \| 1.5 \| 3.9 \| \| \| Major depressive disorder, single episode F32.x \| 10.9 \| 19.8 \| 24.8 \| 18.5 \| 18.6 \| 0.1 \| \| \| Major depressive disorder, recurrent F33.x \| 2.3 \| 5.8 \| 17.7 \| 5.5 \| 4,.9 \| 2.5 \| \| \| Other anxiety disorders F41.9 \| 4.3 \| 5.9 \| 7.5 \| 5.6 \| 5.5 \| 0.4 \| \| \| Panic disorder without agoraphobia F40.0 \| 1.0 \| 0.9 \| 1.1 \| 0.9 \| 1.0 \| 0.7 \| \| \| Sleep disorders not due to a substance or known physiol conditions F51.x \| 0.9 \| 2.0 \| 9.1 \| 2.0 \| 1.6 \| 2.6 \| \| \| Epilepsy and recurrent seizures G40.x \| 0.8 \| 1.6 \| 6.6 \| 1.5 \| 2.2 \| 5.6 \| \| \| Migraine G43.0 \| 6.8 \| 5.7 \| 4.7 \| 5.7 \| 4.7 \| 4.7 \| \| \| Other headache syndromes G44.x \| 2.4 \| 2.2 \| 1.0 \| 2.1 \| 2.4 \| 1.7 \| \| \| Sleep disorders G47.x \| 6.7 \| 13.2 \| 22.0 \| 12.7 \| 12.2 \| 1.5 \| \| \| Essential (primary) hypertension I10.x \| 37.5 \| 61.7 \| 49.8 \| 60.7 \| 61.4 \| 1.5 \| \| \| Hypertensive heart disease I11.x \| 3.6 \| 7.9 \| 18.5 \| 7.7 \| 10.3 \| 9.2 \| \| \| Angina pectoris I20. \| 1.3 \| 3.2 \| 12.5 \| 3.1 \| 4.7 \| 8.5 \| \| \| Myocardial infarction I21,I22 \| 0.8 \| 1.8 \| 9.6 \| 1.8 \| 2.7 \| 5.6 \| \| \| Chronic ischemic heart disease I25.x \| 7.8 \| 22.5 \| 42.0 \| 21.8 \| 27.9 \| 14.2 \| \| \| Nonrheumatic mitral valve disorders I34.x \| 2.5 \| 5.6 \| 15.6 \| 5.5 \| 7.0 \| 6.2 \| \| \| Nonrheumatic aortic valve disorders I35 \| 2.1 \| 5.1 \| 16.1 \| 4.9 \| 7.0 \| 8.8 \| \| \| Other cardiac arrhythmias I49 \| 5.5 \| 10.0 \| 16.9 \| 9.8 \| 11.9 \| 6.8 \| \| \| Heart failure I50.x \| 3.6 \| 14.3 \| 38.1 \| 13.8 \| 21.0 \| 19.0 \| \| \| Cerebrovascular diseases I60-69 \| 5.3 \| 14.1 \| 29.8 \| 13.6 \| 16.8 \| 9.2 \| \| \| Other peripheral vascular diseases I73 \| 2.3 \| 7.2 \| 23.5 \| 7.0 \| 8.7 \| 6.1 \| \| \| Hypotension I95 \| 2.8 \| 2.5 \| 1.6 \| 2.5 \| 2.6 \| 1.0 \| \| \| Other and unspecified disorders of circulatory system I95-I99 \| 1.5 \| 2.0 \| 3.7 \| 1.9 \| 2.6 \| 5.0 \| \| \| Other chronic obstructive pulmonary disease J44 \| 5.5 \| 14.6 \| 30.8 \| 14.2 \| 16.1 \| 5.2 \| \| \| Asthma J45.x \| 7.8 \| 11.1 \| 11.4 \| 10.9 \| 9.0 \| 6.1 \| \| \| Other rheumatoid arthritis M06 \| 1.6 \| 5.2 \| 19.9 \| 4.9 \| 5.2 \| 1.1 \| \| \| Gout M10 \| 2.2 \| 4.4 \| 12.5 \| 4.3 \| 4.7 \| 1.9 \| \| \| Other arthritis M13 \| 1.8 \| 4.0 \| 13.0 \| 3.9 \| 3.6 \| 1.3 \| \| \| Dorsalgia M54.x \| 35.5 \| 54.4 \| 38.8 \| 53.8 \| 42.4 \| 23.0 \| \| \| Neuralgia and neuritis, unspecified M79.2 \| 0.7 \| 1.9 \| 10.9 \| 1.6 \| 1.6 \| 0.2 \| \| \| Pain in limb, hand, foot, fingers and toes M79.6 \| 2.7 \| 4.2 \| 8.4 \| 4.2 \| 3.5 \| 3.9 \| \| \| Stress incontinence (female) (male) N39.3 \| 1.2 \| 2.3 \| 8.2 \| 2.2 \| 2.7 \| 3.0 \| \| \| Other specified urinary incontinence N39.4 \| 1.4 \| 3.7 \| 14.6 \| 3.4 \| 4.2 \| 3.9 \| \| \| Abdominal and pelvic pain R10 \| 11.8 \| 10.9 \| 2.8 \| 10.6 \| 9.4 \| 4.2 \| \| \| Unspecified urinary incontinence R32 \| 2.1 \| 7.5 \| 25.7 \| 6.7 \| 9.1 \| 8.9 \| \| \| Headache R51 \| 5.3 \| 4.8 \| 2.6 \| 4.7 \| 4.2 \| 2.3 \| \| \| Pain, unspecified R52 \| 4.2 \| 18.2 \| 45.3 \| 17.0 \| 15.2 \| 4.8 \| \| \| Senility R54 \| 0.6 \| 1.9 \| 12.2 \| 2.0 \| 2.4 \| 3.4 \| \| \| Transarterial left heart-catheter examination 1-275 \| 1.2 \| 3.1 \| 13.2 \| 3.2 \| 5.0 \| 9.3 \| \| \| Endospopic biopsy upper gastrointestinal tract, bile tract and pankreas 1-440 \| 0.8 \| 3.0 \| 16.4 \| 2.8 \| 3.8 \| 5.7 \| \| \| Diagnostic colonoscpoy 1-650 \| 2.2 \| 4.7 \| 13.5 \| 4,4 \| 4.6 \| 1.1 \| \| \| Number hospitalizations in [t0-365] \| 19.1 \| 63.2 \| 48.9 \| 59.1 \| 68.9 \| 8.3 \| \| \| Aminosalicylic acids and similar agents A07EC \| 0.6 \| 1.8 \| 11.4 \| 1.7 \| 1.5 \| 1.7 \| \| \| Antidiabetics A10 \| 8.0 \| 19.0 \| 32.4 \| 18.7 \| 21.8 \| 7.9 \| \| \| Antithrombotic agents B01 \| 9.0 \| 27.0 \| 48.2 \| 25.9 \| 32.0 \| 13.5 \| \| \| Anti-platet agents, excl. heparin B01AC \| 4.6 \| 13.8 \| 32.1 \| 13.0 \| 19,1 \| 16.8 \| \| \| Acetylsalicylic acid B01AC06 \| 3.7 \| 10.8 \| 27.3 \| 10.0 \| 15.8 \| 17.3 \| \| \| Organic nitrats C01DA \| 1.6 \| 6.2 \| 24.0 \| 5.7 \| 9.0 \| 12.7 \| \| \| High ceiling diuretics C03C \| 4.8 \| 22.2 \| 52.7 \| 20.7 \| 28,.7 \| 18.7 \| \| \| Beta-adrenoreceptor-antagonist C07 \| 19.6 \| 39.3 \| 44.2 \| 38.6 \| 42.2 \| 7.5 \| \| \| Calcium channel blockers C08 \| 9.2 \| 21.5 \| 34.8 \| 20.1 \| 22.3 \| 3.2 \| \| \| ACE-inhibitors, combination C09B \| 6.4 \| 9.7 \| 12.4 \| 9.7 \| 9.1 \| 1.9 \| \| \| Angiotensin-II-antagonists C01C \| 5.4 \| 10.7 \| 19.5 \| 10.5 \| 12.6 \| 6.8 \| \| \| HMG-CoA-reduktase inhibitors C10AA \| 12.2 \| 24.8 \| 32.9 \| 24.3 \| 28.5 \| 9.5 \| \| \| Fibrates C10AB \| 0.5 \| 0.9 \| 4.9 \| 1.0 \| 0.9 \| 0.7 \| \| \| Corticosteroids, systemic H02Bx \| 6.4 \| 16.9 \| 33.3 \| 16.7 \| 14.9 \| 4.9 \| \| \| Immunsuppres-sive agents, other L04AX \| 0.5 \| 1.3 \| 8.4 \| 1.4 \| 1.7 \| 2.5 \| \| \| Non-steroidal agents M01A \| 8.1 \| 41.8 \| 84.6 \| 40.5 \| 32.0 \| 17.8 \| \| \| Analgesics N02 \| 9.4 \| 43.0 \| 82.5 \| 40.9 \| 30.0 \| 23.3 \| \| \| Anticonvulsants N03A \| 1.1 \| 7.0 \| 30.2 \| 5.2 \| 6.5 \| 5.0 \| \| \| Psycholeptics N05 \| 4.5 \| 14.8 \| 35.5 \| 13.2 \| 16.9 \| 10.1 \| \| \| Antipsychotics N05A \| 1.5 \| 6.0 \| 23.9 \| 5.0 \| 7.4 \| 10.3 \| \| \| Benzodiazepin-derivates N05BA \| 1.8 \| 6.0 \| 21.5 \| 5.4 \| 5.9 \| 2.4 \| \| \| Zopiclon N05CF01 \| 0.8 \| 3.0 \| 16.4 \| 2.7 \| 3.9 \| 6.6 \| \| \| Antidepressants N06A \| 1.8 \| 13.0 \| 43.6 \| 10.3 \| 8.7 \| 5.5 \| \| \| Corticosteroids R01AD \| 4.3 \| 3.7 \| 3.2 \| 3.8 \| 3.1 \| 3.5 \| \| \| Products for obstructive airway diseases R03 \| 10.2 \| 18.3 \| 23.4 \| 17.9 \| 19.0 \| 3.0 \| \| \| Selective Beta2-adrenoceptor-agonists R03AC \| 5.1 \| 11.0 \| 21.6 \| 10.7 \| 10.4 \| 1.0 \| \| \| Anticholinergica R03BB \| 1.2 \| 5.0 \| 22.2 \| 4.9 \| 6.4 \| 6.4 \| \| \| Anticonvulsants low dose* \| 0.1 \| 2.8 \| 23.2 \| 1.6 \| 1.5 \| 0.5 \| \| \| Anitdepressants high dose* \| 0.1 \| 2.6 \| 21.8 \| 1.5 \| 1.1 \| 2.7 \| \| \| Antidepressants low dose* \| 0.1 \| 3.3 \| 24.8 \| 1.9 \| 2.0 \| 1.3 \| \| | | | | | |  |
| --- | --- | --- | --- | --- | --- | --- | --- | --- | --- | --- | --- | --- | --- | --- | --- | --- | --- | --- | --- | --- | --- | --- | --- | --- | --- | --- | --- | --- | --- | --- | --- | --- | --- | --- | --- | --- | --- | --- | --- | --- | --- | --- | --- | --- | --- | --- | --- | --- | --- | --- | --- | --- | --- | --- | --- | --- | --- | --- | --- | --- | --- | --- | --- | --- | --- | --- | --- | --- | --- | --- | --- | --- | --- | --- | --- | --- | --- | --- | --- | --- | --- | --- | --- | --- | --- | --- | --- | --- | --- | --- | --- | --- | --- | --- | --- | --- | --- | --- | --- | --- | --- | --- | --- | --- | --- | --- | --- | --- | --- | --- | --- | --- | --- | --- | --- | --- | --- | --- | --- | --- | --- | --- | --- | --- | --- | --- | --- | --- | --- | --- | --- | --- | --- | --- | --- | --- | --- | --- | --- | --- | --- | --- | --- | --- | --- | --- | --- | --- | --- | --- | --- | --- | --- | --- | --- | --- | --- | --- | --- | --- | --- | --- | --- | --- | --- | --- | --- | --- | --- | --- | --- | --- | --- | --- | --- | --- | --- | --- | --- | --- | --- | --- | --- | --- | --- | --- | --- | --- | --- | --- | --- | --- | --- | --- | --- | --- | --- | --- | --- | --- | --- | --- | --- | --- | --- | --- | --- | --- | --- | --- | --- | --- | --- | --- | --- | --- | --- | --- | --- | --- | --- | --- | --- | --- | --- | --- | --- | --- | --- | --- | --- | --- | --- | --- | --- | --- | --- | --- | --- | --- | --- | --- | --- | --- | --- | --- | --- | --- | --- | --- | --- | --- | --- | --- | --- | --- | --- | --- | --- | --- | --- | --- | --- | --- | --- | --- | --- | --- | --- | --- | --- | --- | --- | --- | --- | --- | --- | --- | --- | --- | --- | --- | --- | --- | --- | --- | --- | --- | --- | --- | --- | --- | --- | --- | --- | --- | --- | --- | --- | --- | --- | --- | --- | --- | --- | --- | --- | --- | --- | --- | --- | --- | --- | --- | --- | --- | --- | --- | --- | --- | --- | --- | --- | --- | --- | --- | --- | --- | --- | --- | --- | --- | --- | --- | --- | --- | --- | --- | --- | --- | --- | --- | --- | --- | --- | --- | --- | --- | --- | --- | --- | --- | --- | --- | --- | --- | --- | --- | --- | --- | --- | --- | --- | --- | --- | --- | --- | --- | --- | --- | --- | --- | --- | --- | --- | --- | --- | --- | --- | --- | --- | --- | --- | --- | --- | --- | --- | --- | --- | --- | --- | --- | --- | --- | --- | --- | --- | --- | --- | --- | --- | --- | --- | --- | --- | --- | --- | --- | --- | --- | --- | --- | --- | --- | --- | --- | --- | --- | --- | --- | --- | --- | --- | --- | --- | --- | --- | --- | --- | --- | --- | --- | --- | --- | --- | --- | --- | --- | --- | --- | --- | --- | --- | --- | --- | --- | --- | --- | --- | --- | --- | --- | --- | --- | --- | --- | --- | --- | --- | --- | --- | --- | --- | --- | --- | --- | --- | --- | --- | --- | --- | --- | --- | --- | --- | --- | --- | --- | --- | --- | --- | --- | --- | --- | --- | --- | --- | --- | --- | --- | --- | --- | --- | --- | --- | --- | --- | --- | --- | --- | --- | --- | --- | --- | --- | --- | --- | --- | --- | --- | --- | --- | --- | --- | --- | --- | --- | --- | --- | --- | --- | --- | --- | --- | --- | --- | --- | --- | --- | --- | --- | --- | --- | --- | --- | --- | --- | --- | --- | --- | --- | --- | --- | --- | --- | --- | --- | --- | --- | --- | --- | --- | --- | --- | --- | --- | --- | --- | --- | --- | --- | --- | --- | --- | --- | --- | --- | --- | --- | --- | --- | --- | --- | --- | --- | --- | --- | --- | --- | --- | --- | --- | --- | --- | --- | --- | --- | --- | --- | --- | --- | --- | --- | --- | --- | --- | --- | --- | --- | --- | --- | --- | --- | --- | --- | --- | --- | --- | --- | --- | --- | --- | --- | --- | --- | --- | --- | --- | --- | --- | --- | --- | --- | --- | --- | --- | --- | --- | --- | --- | --- | --- | --- | --- | --- | --- | --- | --- | --- | --- | --- | --- | --- | --- | --- | --- | --- | --- | --- | --- | --- | --- | --- | --- | --- | --- | --- | --- | --- | --- | --- | --- | --- | --- | --- | --- | --- | --- | --- | --- | --- | --- | --- | --- | --- | --- | --- | --- | --- | --- | --- | --- | --- | --- | --- | --- | --- | --- | --- | --- | --- | --- | --- |
|  |  |  |  |  |  |  |

ATC= Anatomical Therapeutic Chemical / Defined Daily Dose Classification*;* ICD= International Classification of Diseases; OPS= official classification for the encoding of operations, procedures and general medical measures

*For definition, see Additional file 6, Table 6
