# Supplementary material for: All-cause mortality in patients with long-term opioid therapy compared with non-opioid analgesics for chronic non-cancer pain: a database study
Source: BMC Med. 2020 Jul 15;18:162. doi: 10.1186/s12916-020-01644-4 (PMC7362543; doi:10.1186/s12916-020-01644-4)
Supplement: Supplementary file 6 — Additional file 6: Table S6. Definition of low dose and high dose anticonvulsants and antidepressants. [file 12916_2020_1644_MOESM6_ESM.docx]

**Additional file 6, Table 6:** **Definition of low dose and high dose anticonvulsants and antidepressants**

**Anticonvulsants,** based on gabapentin equivalence. The low dosage is defined as a daily dosage of less than 600mg/d gabapentin equivalents and a high dosage of

600mg/d or more gabapentin equivalents.

**Standard psychiatric doses as threshold for low/high dosage**

**Active substance ATC code Minimum standard psychatric dose mg/d**

Amitriptyline N06AA09 100

Amitriptylinoxide N06AA09 100

Clomipramine N06AA04 100

Desipramine N06AA01 100

Dibenzepine N06AA08 240

Doxepin N06AA12 100

Imipramine N06AA02 100

Maprotiline N06AA21 100

Nortriptyline N06AA10 50

Trimipramine N06AA06 100

Citalopram N06AB04 20

Escitalopram N06AB10 10

Fluoxetine N06AB03 20

Fluvoxamine N06AB08 100

Paroxetine N06AB05 20

Sertraline N06AB06 50

Moclobemide N06AG02 300

Tranylcypromine N06AF04 20

Venlafaxine N06AX16 75

Duloxetine N06AX21 60

Reboxetine N06AX18 8

Mianserine N06AX03 60

Mirtazapin N06AX11 15

Trazodone N06AX05 200
