# Supplementary material for: All-cause mortality in patients with long-term opioid therapy compared with non-opioid analgesics for chronic non-cancer pain: a database study
Source: BMC Med. 2020 Jul 15;18:162. doi: 10.1186/s12916-020-01644-4 (PMC7362543; doi:10.1186/s12916-020-01644-4)
Supplement: Supplementary file 7 — Additional file 7: Table S7. Sensitivity analysis of predictors of all-cause mortality in the study sample (N = 2813 in non-opioid and N = 2757 in opioid group). [file 12916_2020_1644_MOESM7_ESM.docx]

**Additional file 7, Table 7:** **Sensitivity analysis of predictors of all-cause mortality in the study sample (N=2813 in non-opioid and N=2757 in opioid group)**

| Predictor | Adjusted HR (95%-CI); p-value |
| --- | --- |
| Gender    Male     Female | 1.34 (1.13 – 1.57); <0.0001   Referent |
| Age (per year) | 1.10 (1.10 – 1.12 ); <0.0001 |
| Long-term opioid therapy   Non – opioid therapy | 1.35 (1.14 – 1.58); 0.0004   Referent |
| Duration of drug therapy (per month) | 0.996 (0.995 – 0.996); <0.0001 |
| Comorbidity Index | 1.20 (1.16 – 1.23); <0.0001 |
| Estimated propensity score | 1.70 (1.167 – 2.467); 0.006 |
| Index quarter | 0.998 (0.998 – 0.999); <.0001 |
