# Supplementary material for: All-cause mortality in patients with long-term opioid therapy compared with non-opioid analgesics for chronic non-cancer pain: a database study
Source: BMC Med. 2020 Jul 15;18:162. doi: 10.1186/s12916-020-01644-4 (PMC7362543; doi:10.1186/s12916-020-01644-4)
Supplement: Supplementary file 8 — Additional file 8: Table S8. Predictors of all-cause mortality in patients with < 100 MEQ/. [file 12916_2020_1644_MOESM8_ESM.docx]

**Additional file 8, Table 8:** **Predictors of all-cause mortality in patients with < 100 MEQ/d (N=2 943)**

| **Predictor** | **Adjusted HR**  **(95% CI); p-value** |
| --- | --- |
| Gender    Male      Female | 1.28 (1.08 - 1.47); <0.001  Referent |
| Age (per year) | 1.09 (1.08 -1.10); <0.0001 |
| Long-term opioid therapy    Non – opioid therapy | 1.64 (1.43 – 1.89); <0.0001   Referent |
| Duration of drug therapy (per month) | 0.99 (0.99 - 0.99); <0.0001 |
| Comorbidity Index | 1.19 (1.16- 1.22); <0.0001 |
| Estimated propensity score | 1.26 (0.92 -1.73); 0.15 |
| Index quarter | 0.99 (0.99 – 0.99); <0.0001 |
| Treatment duration | 0.99 (0.99- 0.99); <0.0001 |
