# Supplementary material for: All-cause mortality in patients with long-term opioid therapy compared with non-opioid analgesics for chronic non-cancer pain: a database study
Source: BMC Med. 2020 Jul 15;18:162. doi: 10.1186/s12916-020-01644-4 (PMC7362543; doi:10.1186/s12916-020-01644-4)
Supplement: Supplementary file 9 — Additional file 9: Table S9. Predictors of all-cause mortality in patients with ≥100 MEQ/d. [file 12916_2020_1644_MOESM9_ESM.docx]

Additional file 9, Table 9: Predictors of all-cause mortality in patients with ≥100 MEQ/d (N=189)

| **Predictor** | **Adjusted HR**  **(95% CI); p-value** |
| --- | --- |
| Gender    Male     Female | 1.28 (1.12 -1∙47); <0.001  Referent |
| Age (per year) | 1.09 (1.09 - 1∙10); <0.0001 |
| Long-term opioid therapy    Non – opioid therapy | 1.59 (1.38 - 1∙83); <0.0001   Referent |
| Duration of drug therapy (per month) | 0.99 (0.99 - 0∙99); <0.0001 |
| Comorbidity Index | 1.19 (1.16 -1∙23); <0.0001 |
| Estimated propensity score | 1.32 (0.96 - 1∙80); 0.09 |
| Index quarter | 0.99 (0.99 - 0∙99); <0.0001 |
| Treatment duration | 0.99 (0.99- 0∙99); <0.0001 |
